# Acute respiratory distress syndrome: prevention and early recognition

**DOI:** 10.1186/2110-5820-3-11

**Published:** 2013-04-24

**Authors:** Candelaria de Haro, Ignacio Martin-Loeches, Eva Torrents, Antonio Artigas

**Affiliations:** 1 Critical Care Centre, Hospital de Sabadell, Corporació Sanitària i Universitària Parc Taulí, Universitat Autònoma de Barcelona, CIBER Enfermedades Respiratorias, Sabadell, Spain

## Abstract

Acute respiratory distress syndrome (ARDS) is common in critically ill patients admitted to intensive care units (ICU). ARDS results in increased use of critical care resources and healthcare costs, yet the overall mortality associated with these conditions remains high. Research focusing on preventing ARDS and identifying patients at risk of developing ARDS is necessary to develop strategies to alter the clinical course and progression of the disease. To date, few strategies have shown clear benefits. One of the most important obstacles to preventive interventions is the difficulty of identifying patients likely to develop ARDS. Identifying patients at risk and implementing prevention strategies in this group are key factors in preventing ARDS. This review will discuss early identification of at-risk patients and the current prevention strategies.

## Review

### Introduction

Acute respiratory distress syndrome (ARDS) is common in critically ill patients admitted to intensive care units (ICU).

### Multiple hit model for ALI/ARDS development

**Figure 1:**
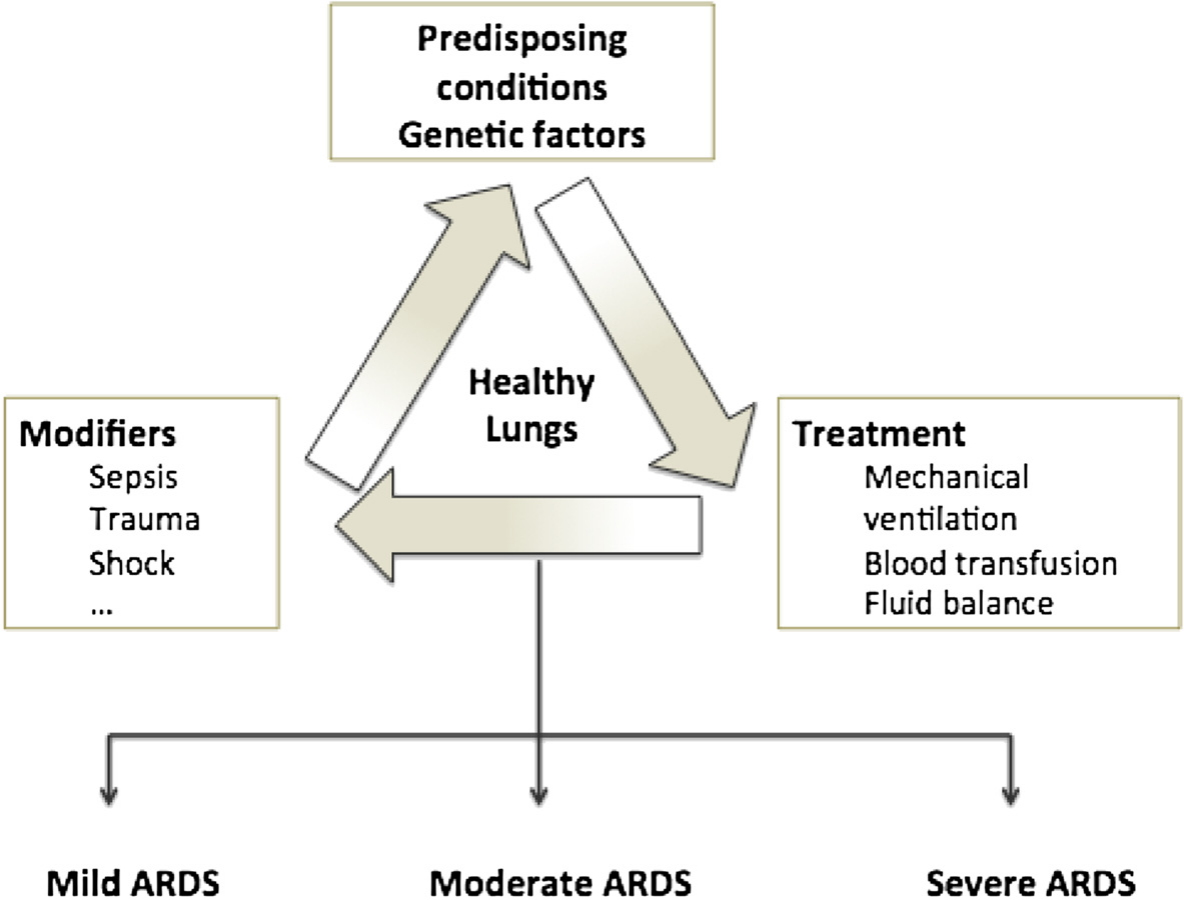
**Multiple-hit model.** A chain reaction of predisposing conditions and multiple hits (modifiers factors and treatments) in healthy lungs can develop mild, moderate or severe ARDS.

Previous studies support a two-hit model of ARDS development in which exposure to pertinent risk factors modifies the development and expression of ARDS in a host with predisposing conditions [1-3]. However, beyond the development of ARDS in a susceptible host, theories of the pathogenesis of this condition should explain progression from an initial lung injury to mild ARDS or from mild or moderate to severe ARDS. To this end, a chain reaction based on multiple hits can be involved in the pathogenesis of ARDS development and/or the progression of severity [4]. Host predisposing conditions act as a first hit in healthy lungs, where multiple hit can induce ARDS. In the absence of these predisposing conditions, the probability that the other hits would result in ARDS is lower. Whether the disease progresses from an initial injury to ARDS or continues to progress from mild-moderate ARDS to severe ARDS probably depends on a chain reaction based on these multiple hits (Figure 1).

Hudson et al. [5] found the presence of one or more of eight clinical conditions (sepsis, aspiration, drug overdose, near-drowning, pulmonary contusion, multiple transfusions, multiple fractures, head trauma) predicted whether ARDS develops with 79% sensitivity but only 26% specificity. Fowler et al. [6] found pneumonia, aspiration, and disseminated intravascular coagulation were the strongest predictors but only 7% of the patients with one or more predisposing factors developed ALI. Gong et al. [7] showed the risk of ARDS increased with a pulmonary etiology of injury, hematologic failure, transfusion of eight or more units of packed red blood cells, respiratory rate > 33 breaths/min, hematocrit > 37.5%, arterial pH < 7.33, albumin ≤ 2.3 g/dL, and transfer from another hospital. In another study in non-ICU hospitalized patients, Ferguson et al. [8] found that pulmonary risk conditions had a higher rate of progression to ARDS than nonpulmonary conditions, but shock was the strongest predictor. Therefore, by halting this unvirtous circle might help to stop the progression to ARDS.

### Towards prevention. Early recognition

#### Identification of patients at risk

The first obstacle to preventing ARDS is identifying patients at risk of developing ARDS [9]. Many authors have proposed clinically detectable predisposing factors and even scores to identify patients at risk early in the course of the disease. Implementing preventive measures requires an algorithm for early detection. Different algorithms have been proposed. Trillo-Alvarez et al. [10] developed an ALI prediction score, the lung injury prediction score (LIPS), that identifies patients at high risk for ALI before ICU admission. Risk factors are divided into predisposing conditions (sepsis, shock, pneumonia, aspiration, trauma, and high risk-surgery) and risk modifiers (obesity, alcohol abuse, diabetes, hypoalbuminemia, acidosis, tachypnea, and oxygen supplementation). Gajic et al. [11] validated this score in a prospective, multicenter, observational study, demonstrating that the LIPS model discriminates efficiently between patients who have a low risk of developing ARDS and those with high risk (AUC = 0.8), while maintaining an appropriate sensitivity for screening (negative predictive value (NPV) = 0.97).

In 2010, Thakur et al. [12] reported a cohort study designed to identify patients at risk early before ARDS develops, using an electronic medical record that facilitates early recognition of specific criteria, based on a previously validated ARDS sniffer (NPV = 0.99, 95% confidence interval (CI) 0.98-1.00) [13]. They identified patients at risk by the LIPS score and recorded hospital-acquired exposures that may modify the risk of ARDS and its impact on subgroups of high-risk patients. Levitt et al. [14] proposed the term “early ALI” (EALI). EALI identified patients progressing to ARDS with a sensitivity of 73% and a specificity of 79%. The combination of early clinical recognition and predictive scores could help in the detection of patients at-risk and in the early treatment or implementation of preventive strategies.

#### Biomarkers

Identifying a biomarker that predicts the development of ARDS or progression of severity could be helpful. The ideal biomarker would be easy and safe to collect, easily measured and reproducible, highly sensitive and specific in predicting clinical outcome, and have a defined role in the pathogenesis of ARDS [15]. Unfortunately, no single biomarker is currently specific or sensitive enough to be incorporated into routine clinical practice.

Volatile organic compounds in exhaled air may differ between diseases. Bos et al. [16,17] conducted two studies where they discriminated between mechanically ventilated patients with and without ARDS on the basis of a breathprint obtained by analyzing exhaled air through eNose technology. This breathprint distinguished between patients with ARDS and those without as accurately as PaO_2_/FiO_2_ assessment. Noninvasive analysis of exhaled air may help to identify a biomarker to detect ARDS in an early stage, before clinical signs; however, further studies are needed before this approach can be incorporated into routine clinical practice.

Other biomarkers in plasma or bronchoalveolar lavage fluid have been studied in patients with ARDS. A recent trial in critically ill patients demonstrated that higher levels in plasma of angiopoetin-2 were significantly associated with increased development of ALI (odds ratio (OR) 2.4; 95% CI 1.3-4.2) [18].

Furthermore, the association of angiopoetin-2 levels and the LIPS improved the AUC to 0.84. Because there is no ideal biomarker to help us in the early detection of ARDS, the association between biomarkers and different scoring systems based on clinical data or diagnostic tests could improve prediction scores [19,20].

#### Genetic predisposition

There is some evidence that genetic factors predispose to the development of ARDS and poor prognosis. Copland et al. [21] used an experimental model of acute ventilator-induced lung injury (VILI) to determine which genes changed their expression depending on inspiratory volumes, and they identified various genes with overexpression. One of the most important studies is from Grigoryev et al. [22], who identified 85 genes important in the pathogenesis and different biologic processes involved in the development of ARDS.

Nevertheless, at present, we cannot identify a single gene responsible for a high susceptibility to ARDS. Some studies are currently trying to evaluate the role of single nucleotide polymorphism (SNPs) and haplotypes in order to better detect patients at ARDS risk. These studies could help to define new therapeutic targets, new approaches to treatment, and individual indicators of predisposition to developing the disease that might enable the development of effective preventive strategies.

#### Pathogen virulence

Defining risk factors associated with the development of ARDS in patients with an infectious disease is challenging because virulence factors of different pathogens have been implicated in causing lung damage. Bacterial products are directly toxic to the lung epithelium, independent of host inflammatory responses and other factors [23]. Kojicic et al. [24] recently found that the risk of hospitalized patients with infectious pneumonia developing ALI varied with the pulmonary pathogens.

#### Preventive measures

Beyond the etiology, certain modifiable external factors can accelerate the development of ARDS.

#### Mechanical ventilation

**Lung protective ventilation** Lung-protective mechanical ventilation strategies are the only supportive therapy that clearly improve survival in patients with ARDS. However, mechanical ventilation can lead to lung injury by different mechanisms, with subsequent diffuse alveolar damage, pulmonary edema, and local production of inflammatory mediators. This circumstance is known as VILI.

Tremblay et al. [25] showed that mechanical ventilation with high volumes and without positive end-expiratory pressure (PEEP) could increase tumor necrosis factor (TNF-alpha) and interleukin-6 (IL-6) concentrations in bronchoalveolar lavage fluid, whereas cytokine levels are lower if protective mechanical ventilation is used. Amato et al. [26] demonstrated lower mortality in patients ventilated with a limited tidal volume to avoid overdistension. A prospective, multicenter trial (ARDS Network) published in 2000 confirmed that mechanical ventilation with lower tidal volume (6 mL/Kg predicted body weight (PBW)) resulted in an increase in the number of ventilator-free days and a reduction of in-hospital mortality [27].

Mechanical ventilation is an important factor in the treatment of ARDS, but is it important in prevention? Does mechanical ventilation work as a risk modifier in patients at risk? Could we help to prevent the development of ARDS by applying protective mechanical ventilation in patients at risk?

In a recent study, our group found that septic patients without ARDS ventilated with a protective strategy using a plateau pressure < 30 cmH_2_0 had better outcomes and a lower incidence of ARDS than those ventilated without this limit on plateau pressure [28]. Thus, it may be beneficial to implement protective ventilation strategies from the start of mechanical ventilation, not only when ARDS appears. It remains to be determined whether only plateau pressure should be targeted or whether tidal volume should be targeted too. In our opinion, preventive strategies also should include protective ventilation with low tidal volume in patients at risk, and some studies support this approach. In 2004, Gajic et al. [29] reported that in ventilated patients without ARDS large tidal volumes (11.4 mL/kg PBW for women and 10.4 mL/kg PBW for men) were independently associated with the development of ARDS. In a prospective trial, Determann et al. [30] randomized patients who required mechanical ventilation to receive low tidal volume (6 mL/kg PBW) or conventional tidal volume (10 mL/kg PBW); the trial was stopped prematurely because more patients in the conventional tidal-volume group developed ARDS than in the lower tidal-volume group (13.5 vs. 2.6; *p* = 0.01).

Interestingly, the use of protective ventilation in “healthy lungs” seems to be a field for further research. A recently published meta-analysis, including eight trials that evaluated two types of mechanical ventilation in patients with uninjured lungs in the operating room, demonstrated a decrease in lung injury development, pulmonary infection, and atelectasis using lower tidal volumes and high PEEP [31]. These results support the previous meta-analysis that demonstrated only an attenuation of the development of lung injury, conducted with trials that included patients in operating room and ICU [32].

No concrete ventilation strategies have been established for patients without ARDS who require mechanical ventilation. We think that ventilation with low tidal volumes and plateau pressure < 30 cmH_2_O is a reasonable prevention strategy of ARDS. Side effects associated with low tidal volumes seem to be minimal. However, based on a review by Schultz et al. [33], most studies favoring low tidal volume ventilation in patients without ARDS measured inflammatory mediators instead of clinical outcomes, so prospective trials should be done to evaluate the clinical impact of this ventilatory strategy.

#### PEEP strategies

The use of prophylactic PEEP in patients without ARDS is controversial. Various authors have demonstrated that prophylactic PEEP decreases the incidence of ARDS and PEEP may protect the lung after different types of insults. In the 1970s it was demonstrated that intraoperative use of PEEP reduced ARDS development [34] and the use of early prophylactic PEEP reduced the incidence of ARDS [35]. Other studies demonstrated that the use of certain amount of PEEP reduced the intensity of lung injury from different aggressions, as Dreyfuss et al. showed in 1988 with the reduction of edema and preservation of the normal structural aspect of alveolar epithelium using high levels of PEEP [36,37]. However, other studies have refuted this effect. Pepe et al. conducted a trial where found that the early application of PEEP in high-risk patients had no effect on the incidence of ARDS [38]. Manzano et al. [39] conducted a randomized trial where nonhypoxemic patients without lung injury received 5 cmH_2_O or 8 cmH_2_O PEEP versus no PEEP. They found no differences between groups in the development of ARDS, but the proportion of patients who developed hypoxemia was significantly higher in the no-PEEP group and the PEEP group developed less ventilator-associated pneumonia (relative risk (RR) 0.37; 0.15-0.84).

The use of higher tidal volumes, based on older strategies, arose from a need to prevent atelectasis. PEEP strategies are now better understood and the risk of over distension better appreciated. So, this need has lessened.

### Supportive treatments

#### Fluid balance

Fluid balance is an important risk modifier in the development of ARDS. Pulmonary edema is largely due to increased capillary permeability but is exacerbated by increased hydrostatic pressures and oncotic pressure falls [40]. The first reports about fluid strategies date from 1987, when Simmons et al. [41] observed that outcomes in ARDS patients were better if they lost body weight and had a lower fluid balance. Sakr et al. [42] in a cohort observational study showed that a higher mean fluid balance was independently associated with increased mortality in patients with ARDS. In 2006, the ARDS Network published the results of a prospective, randomized trial comparing conservative and liberal fluid strategies in patients with ARDS. The authors included patients after 48 hours of ICU admission and with systolic blood pressure > 60 mmHg. The primary outcome (60-day mortality) did not differ between groups, but the conservative group had better oxygenation index and lung injury scores, as well as more ventilator-free days [43]. A limitation of this study is the exclusion of hemodynamically unstable patients that difficult the generalization of the results to all patients with ARDS. On the other hand, Murphy et al. [44] showed in a retrospective analysis of patients with septic shock and ARDS that an adequate initial fluid resuscitation during the first 2 consecutive days coupled with conservative fluid management after that was associated with the lowest mortality. Despite the above, no randomized trials have been done evaluating a two-phase fluid management strategy that could confirm this strategy. Thus, in patients with established ARDS, it seems that a conservative fluid strategy should be used, but it is difficult to know the role of fluid balance in the absence of ARDS.

#### Sepsis management

Sepsis precipitates ARDS in 25% to 40% of cases, and the risk increases if a systemic inflammatory response, shock, or organ dysfunction is present. The most frequent etiology is pneumonia, followed by nonpulmonary infections [45]. There is no specific preventive treatment against the development of ARDS in patients with sepsis. Novel therapies are being studied, but no promising results have been reported. It seems that early detection of patients with sepsis who are at risk of developing ARDS is one way to achieve better results in the earliest phase. Indeed, one of the most important preventive strategies is to ensure adequate management of sepsis, including source control and early appropriate antibiotic therapy [46,47].

#### Restrictive transfusion

Several studies have demonstrated an association between the transfusion of blood products and ARDS. Zilberberg et al. [48] showed that more patients who developed ARDS had received red cell transfusions than those who did not develop ARDS; moreover, they found that transfusion of greater amounts increased the risk of developing ARDS. Many authors have since confirmed this association [7,45,49,50].

Transfusion-related acute lung injury (TRALI) is defined as new ARDS during or within 6 hours after an infusion of one or more plasma-containing or plasma-derived blood products [51].

The neutrophil has been postulated that is the responsible cell in the pathogenesis of TRALI. In 1985 Popovsky et al. published a sequence of patients who developed TRALI after blood transfusion and demonstrated the presence of leukocyte antibodies and antibodies anti-HLA in a high percentage of blood donators. It was thought that donors’ antibodies were responsible of TRALI, but some transfused patients did not develop TRALI despite the presence of leukocyte antibodies [52].

Silliman et al. proposed a nonimmune model based on two hits: first, a lung injury in the lung epithelium causes sequestration of neutrophils in the lungs, and second, the transfusion of blood storage causes liberation of cytotoxic factors and capillary damage. This concept is because blood storage products are correlated with a high development of transfusion reactions, and factors responsible of these reactions increase with time of storage [53].

The activated neutrophils release oxygen radicals and other elements that damage the lung endothelium. Despite this, there are clinical and experimental evidence of TRALI without neutrophil primo activation, but it is the underlying disease that activates the endothelium and after that neutrophils are recruited in lung capillaries.

These theories of pathogenesis suggest different preventive strategies. Several studies have demonstrated worse outcomes after donation from multiparous women, because the likelihood of HLA and HNA immunization increases with the number of pregnancies [54-56]. Because of this, many authors suggest the restriction of women donors [57-59]. Although it could seem disproportioned, the option of the screening of donors is expensive and not available in all sites.

The strategy of reduction the time of blood storage seems reasonable to prevent nonimmune TRALI. Another option is the leukoreduction, reducing the activity of preactivated neutrophils. It has been introduced in many countries [60]. An adequate policy of transfusion focused on less transfusion is perhaps the most important preventive strategy [61].

#### Specific strategies

Many trials are underway to test whether different pharmacologic treatments have beneficial effects, but many pharmacologic therapies that prove effective in animal models fail in human studies. To date, there is no specific pharmacologic treatment for the prevention of ALI/ARDS.

#### Activated protein C

Activated protein C (APC) has several anti-inflammatory effects that may reduce the likelihood and severity of lung injury. In ARDS, acute inflammation in pulmonary compartments is characterized by fibrin formation, cytokine production, and protein leakage into the alveolar space due to increased capillary barrier permeability and neutrophil migration. All of these are potential targets of recombinant human APC. Local administration of APC would be a good treatment strategy in ARDS, avoiding the bleeding complications associated with systemic administration.

It might be feasible to administer APC to ARDS patients by inhalation. Administering inhaled APC to mice significantly reduced lipopolysaccharide-induced pulmonary inflammation [62]. Another experimental study tested the preventive effect of inhaled APC against lung injury in mice and found a reduction in lung injury induced by mechanical ventilation in the group that received inhaled APC [63].

Nevertheless, the evidence supporting the use of APC is not strong enough to recommend it for the prevention of ARDS. We need more studies.

#### Mesenchymal stem cells

Recent studies using experimental models of ALI have demonstrated that the transplantation of mesenchymal stem cells (MSCs) improves the regeneration of lung tissue [64]. The benefits MSCs are derived not only from the incorporation of these cells in the damaged lung, but also from their interaction with damaged lung cells and immunologic modulation. Most of these studies administered MSCs as a pretreatment; one study in rats showed pretreatment with MSCs reduced VILI [65]. However, the use of MSCs is still highly experimental and more studies are necessary before they can be applied as a treatment or prevention strategy.

#### Other treatments

**Figure 2:**
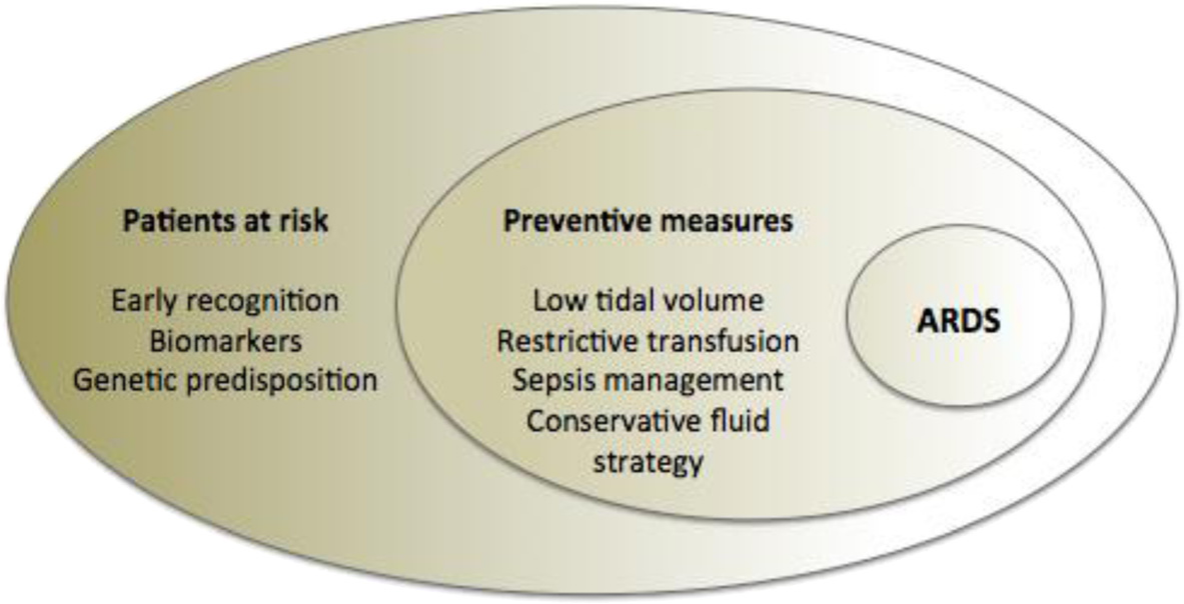
Preventive approaches to ARDS.

Aspirin, statins, inhaled corticosteroids, and beta-2 adrenergic agonists have been tested in experimental studies but have yet to show promising results in human patients. More studies are necessary before these drugs can be incorporated into preventive strategies [66]. Although there is still a long way to go in the ARDS prevention field, some new strategies and preliminary studies promise to improve the early identification and early intervention to prevent the progression of the disease (Figure 2).

## Conclusions

ARDS is a common and devastating complication after acute illness or injury, and it results in high morbidity, mortality, and healthcare costs. Lung-protective ventilation and appropriate sepsis management seems to be the only strategies proven to improve outcomes in ARDS patients; preventive strategies are a new field of research aiming to avoid the progression of the disease. To date, few preventive strategies have shown clear benefits. The combination of early clinical recognition and predictive scores could help to identify patients at risk and those who might progress to mild, moderate, or severe ARDS. The titration of tidal volume and the duration of mechanical ventilation in patients without ARDS seem to be the best strategy for prevention.

## Competing interests

The authors declare that they have no competing interests.

## Authors’ contributions

All authors contributed to the drafting of the manuscript and approved the final version.

## References

[B1] Matthay MA Zimmerman GA Esmon C Bhattacharya J Coller B Doerschuk CM Floros J Gimbrone MA Hoffman E Hubmayr RD Leppert M Matalon S Munford R Parsons P Slutsky AS Tracey KJ Ward P Gail DB Harabin AL Future research directions in acute lung injury: summary of a National Heart, Lung, and Blood Institute working group Am J Respir Crit Care Med 2003 3 1027 1035 10.1164/rccm.200208-966WS 12663342

[B2] Charles PE Tissières P Barbar SD Croisier D Dufour J Dunn-Siegrist I Chavanet P Pugin J Mild-stretch mechanical ventilation upregulates toll-like receptor 2 and sensitizes the lung to bacterial lipopeptide Crit Care 2011 3 4 R181 10.1186/cc10330 21794115 PMC3387624

[B3] Villar J Cabrera N Casula M Flores C Valladares F Muros M Blanch L Slutsky AS Kacmarek RM Mechanical ventilation modulates Toll-like receptor signaling pathway in a sepsis-induced lung injury model Intensive Care Med 2010 3 6 1049 1057 10.1007/s00134-010-1799-3 20397011

[B4] Pavord ID Birring SS Berry M Green RH Brightling CE Wardlaw AJ Multiple inflammatory hits and the pathogenesis of severe airway disease Eur Respir J 2006 3 5 884 888 16707390 10.1183/09031936.06.00128105

[B5] Hudson LD Milberg JA Anardi D Maunder RJ Clinical risks for development of the acute respiratory distress syndrome Am J Respir Crit Care Med 1995 3 293 301 10.1164/ajrccm.151.2.7842182 7842182

[B6] Fowler AA Hamman RF Good JT Benson KN Baird M Eberle DJ Petty TL Hyers TM Adult respiratory distress syndrome: risk with common predispositions Ann Intern Med 1983 3 593 597 10.7326/0003-4819-98-5-593 6846973

[B7] Gong MN Thompson BT Williams P Pothier L Boyce PD Christiani DC Clinical predictors of and mortality in acute respiratory distress syndrome: potential role of red cell transfusion Crit Care Med 2005 3 1191 1198 10.1097/01.CCM.0000165566.82925.14 15942330

[B8] Ferguson ND Frutos-Vivar F Esteban A Gordo F Honrubia T Peñuelas O Algora A García G Bustos A Rodríguez I Clinical risk conditions for acute lung injury in the intensive care unit and hospital ward: a prospective observational study Crit Care 2007 3 R96 10.1186/cc6113 17784960 PMC2556739

[B9] Levitt JE Matthay MA Clinical review: early treatment of acute lung injury - paradigm shift toward prevention and treatment prior to respiratory failure Crit Care 2012 3 223 10.1186/cc11144 22713281 PMC3580596

[B10] Trillo-Alvarez C Cartin-Ceba R Kor DJ Kojicic M Kashyap R Thakur S Thakur L Herasevich V Malinchoc M Gajic O Acute lung injury prediction score: derivation and validation in a population-based sample Eur Respir J 2011 3 604 609 10.1183/09031936.00036810 20562130

[B11] Gajic O Dabbagh O Park PK Adesanya A Chang SY Hou P Anderson H Hoth JJ Mikkelsen ME Gentile NT Gong MN Talmor D Bajwa E Watkins TR Festic E Yilmaz M Iscimen R Kaufman DA Esper AM Sadikot R Douglas I Sevransky J Malinchoc M Early identification of patients at risk of acute lung injury: evaluation of lung injury prediction score in a multicenter cohort study Am J Respir Crit Care Med 2011 3 462 470 10.1164/rccm.201004-0549OC 20802164 PMC3056224

[B12] Thakur SJ Trillo-Alvarez CA Malinchoc MM Kashyap R Thakur L Ahmed A Reriani MK Cartin-Ceba R Sloan JA Gajic O Towards the prevention of acute lung injury: a population based cohort study protocol BMC Emerg Med 2010 3 8 10.1186/1471-227X-10-8 20420711 PMC2873575

[B13] Herasevich V Yilmaz M Khan H Hubmayr RD Gajic O Validation of an electronic surveillance system for acute lung injury Intensive Care Med 2009 3 1018 1023 10.1007/s00134-009-1460-1 19280175 PMC2730460

[B14] Levitt JE Bedi H Calfee CS Gould MK Matthay MA Identification of early acute lung injury at initial evaluation in an acute care setting prior to the onset of respiratory failure Chest 2009 3 936 943 10.1378/chest.08-2346 19188549 PMC2758305

[B15] Moriates C Maisel A The utility of biomarkers in sorting out the complex patient Am J Med 2010 3 393 399 10.1016/j.amjmed.2009.07.034 20399312

[B16] Bos LDJ Fens N van der Schee MP Sterk P Schultz MJ Fast assessment of ALI/ARDS in the ICU using exhaled breath analysis Am J Respir Crit Care Med 2010 3 A2583

[B17] Bos LDJ Sterk PJ Schuktz MJ Exhaled breath analysis in the diagnosis of acute lung injury Am J Respir Crit Care Med 2011 3 A1163

[B18] Agrawal A Matthay MA Kangelaris KN Stein J Chu JC Imp BM Cortez A Abbott J Liu KD Calfee CS Plasma angiopoietin-2 predicts the onset of acute lung injury in critically Ill patients Am J Respir Crit Care Med 2013 [Epub ahead of print] 10.1164/rccm.201208-1460OC PMC3678110 23328529

[B19] Ware LB Koyama T Billheimer DD Wu W Bernard GR Thompson BT Brower RG Standiford TJ Martin TR Matthay MA NHLBI ARDS Clinical Trials Network Prognostic and pathogenetic value of combining clinical and biochemical indices in patients with acute lung injury Chest 2010 3 2 288 296 10.1378/chest.09-1484 19858233 PMC2816641

[B20] Calfee CS Ware LB Glidden DV Eisner MD Parsons PE Thompson BT Matthay MA National Heart, Blood, and Lung Institute Acute Respiratory Distress Syndrome Network Use of risk reclassification with multiple biomarkers improves mortality prediction in acute lung injury Crit Care Med 2011 3 4 711 717 10.1097/CCM.0b013e318207ec3c 21283009 PMC3260884

[B21] Copland IB Kavanagh BP Engelberts D McKerlie C Belik J Post M Early changes in lung gene expression due to high tidal volume Am J Respir Crit Care Med 2003 3 9 1051 1059 Epub 2003 Jun 19 10.1164/rccm.200208-964OC 12816737

[B22] Grigoryev DN Finigan JH Hassoun P Garcia JG Science review: searching for gene candidates in acute lung injury Crit Care 2004 3 6 440 447 Epub 2004 Jun 30 10.1186/cc2901 15566614 PMC1065043

[B23] Martin TR Direct lung injury by bacteria: clarifying the tools of the trade Crit Care Med 2004 3 11 2360 2361 15640664 10.1097/01.ccm.0000146135.19642.72

[B24] Kojicic M Li G Hanson AC Lee KM Thakur L Vedre J Ahmed A Baddour LM Ryu JH Gajic O Risk factors for the development of acute lung injury in patients with infectious pneumonia Crit Care 2012 3 2 R46 10.1186/cc11247 22417886 PMC3568742

[B25] Tremblay L Valenza F Ribeiro SP Li J Slutsky AS Injurious ventilatory strategies increase cytokines and c-fos m-RNA expression in an isolated rat lung model J Clin Invest 1997 3 944 952 10.1172/JCI119259 9062352 PMC507902

[B26] Amato MB Barbas CS Medeiros DM Magaldi RB Schettino GP Lorenzi-Filho G Kairalla RA Deheinzelin D Munoz C Oliveira R Takagaki TY Carvalho CR Effect of a protective-ventilation strategy on mortality in the acute respiratory distress syndrome N Engl J Med 1998 3 347 354 10.1056/NEJM199802053380602 9449727

[B27] The Acute Respiratory Distress Syndrome Network Ventilation with lower tidal volumes as compared with traditional tidal volumes for acute lung injury and the acute respiratory distress syndrome. The Acute Respiratory Distress Syndrome Network N Engl J Med 2000 3 1301 1308 10.1056/NEJM200005043421801 10793162

[B28] Martin-Loeches I de Haro C Dellinger RP Ferrer R Phillips GS Levy MM Artigas A Effectiveness of inspiratory pressure-limited approach to mechanical ventilation in septic patients Eur Respir J 2012 Apr 20. [Epub ahead of print] 10.1183/09031936.00221611 22523366

[B29] Gajic O Dara SI Mendez JL Adesanya AO Festic E Caples SM Rana R St Sauver JL Lymp JF Afessa B Hubmayr RD Ventilator-associated lung injury in patients without acute lung injury at the onset of mechanical ventilation Crit Care Med 2004 3 1817 1824 10.1097/01.CCM.0000133019.52531.30 15343007

[B30] Determann RM Royakkers A Wolthuis EK Vlaar AP Choi G Paulus F Hofstra JJ de Graaff MJ Korevaar JC Schultz MJ Ventilation with lower tidal volumes as compared with conventional tidal volumes for patients without acute lung injury: a preventive randomized controlled trial Crit Care 2010 3 R1 10.1186/cc8230 20055989 PMC2875503

[B31] Hemmes SN Neto AS Schultz MJ Intraoperative ventilatory strategies to prevent postoperative pulmonary complications: a meta-analysis Curr Opin Anaesthesiol 2013 3 2 126 133 10.1097/ACO.0b013e32835e1242 23385321

[B32] Serpa Neto A Cardoso SO Manetta JA Pereira VG Espósito DC Pasqualucci Mde O Damasceno MC Schultz MJ Association between use of lung-protective ventilation with lower tidal volumes and clinical outcomes among patients without acute respiratory distress syndrome: a meta-analysis JAMA 2012 3 16 1651 1659 10.1001/jama.2012.13730 23093163

[B33] Schultz MJ Haitsma JJ Slutsky AS Gajic O What tidal volumes should be used in patients without acute lung injury? Anesthesiology 2007 3 1226 1231 10.1097/01.anes.0000267607.25011.e8 17525599

[B34] Schmidt GB O'Neill WW Kotb K Hwang KK Bennett EJ Bombeck CT Continuous positive airway pressure in the prophylaxis of the adult respiratory distress syndrome Surg Gynecol Obstet 1976 3 4 613 618 785646

[B35] Weigelt JA Mitchell RA Snyder WH 3rd Early positive end-expiratory pressure in the adult respiratory distress syndrome Arch Surg 1979 3 4 497 501 10.1001/archsurg.1979.01370280151024 373705

[B36] Dreyfuss D Soler P Basset G Saumon G High inflation pressure pulmonary edema. Respective effects of high airway pressure, high tidal volume, and positive end-expiratory pressure Am Rev Respir Dis 1988 3 5 1159 1164 10.1164/ajrccm/137.5.1159 3057957

[B37] Ruiz-Bailén M Fernández-Mondéjar E Hurtado-Ruiz B Colmenero-Ruiz M Rivera-Fernández R Guerrero-López F Vázquez-Mata G Immediate application of positive-end expiratory pressure is more effective than delayed positive-end expiratory pressure to reduce extravascular lung water Crit Care Med 1999 3 2 380 384 10.1097/00003246-199902000-00046 10075064

[B38] Pepe PE Hudson LD Carrico CJ Early application of positive end-expiratory pressure in patients at risk for the adult respiratory-distress syndrome N Engl J Med 1984 3 5 281 286 10.1056/NEJM198408023110502 6377071

[B39] Manzano F Fernández-Mondéjar E Colmenero M Poyatos ME Rivera R Machado J Catalán I Artigas A Positive-end expiratory pressure reduces incidence of ventilator-associated pneumonia in nonhypoxemic patients Crit Care Med 2008 3 8 2225 2231 10.1097/CCM.0b013e31817b8a92 18664777

[B40] Wiedemann HP Wheeler AP Bernard GR Thompson BT Hayden D deBoisblanc B Connors AF Jr Hite RD Harabin AL National Heart, Lung, and Blood Institute Acute Respiratory Distress Syndrome (ARDS) Clinical Trials Network Comparison of two fluid-management strategies in acute lung injury N Engl J Med 2006 3 24 2564 2575 16714767 10.1056/NEJMoa062200

[B41] Simmons RS Berdine GG Seidenfeld JJ Prihoda TJ Harris GD Smith JD Gilbert TJ Mota E Johanson WG Fluid balance and the adult respiratory distress syndrome Am Rev Respir Dis 1987 3 924 929 3565940 10.1164/arrd.1987.135.4.924

[B42] Sakr Y Vincent JL Reinhart K Groeneveld J Michalopoulos A Sprung CL Artigas A Ranieri VM High tidal volume and positive fluid balance are associated with worse outcome in acute lung injury Chest 2005 3 3098 3108 10.1378/chest.128.5.3098 16304249

[B43] Wiedemann HP Wheeler AP Bernard GR Thompson BT Hayden D deBoisblanc B Connors AF Jr Hite RD Harabin AL Comparison of two fluid-management strategies in acute lung injury N Engl J Med 2006 3 24 2564 2575 Epub 2006 May 21 16714767 10.1056/NEJMoa062200

[B44] Murphy CV Schramm GE Doherty JA Reichley RM Gajic O Afessa B Micek ST Kollef MH The importance of fluid management in acute lung injury secondary to septic shock Chest 2009 3 1 102 109 Epub 2009 Mar 24 10.1378/chest.08-2706 19318675

[B45] Iscimen R Cartin-Ceba R Yilmaz M Khan H Hubmayr RD Afessa B Gajic O Risk factors for the development of acute lung injury in patients with septic shock: an observational cohort study Crit Care Med 2008 3 1518 1522 10.1097/CCM.0b013e31816fc2c0 18434908

[B46] Ferrer R Artigas A Levy MM Blanco J González-Díaz G Garnacho-Montero J Ibáñez J Palencia E Quintana M de la Torre-Prados MV Improvement in process of care and outcome after a multicenter severe sepsis educational program in Spain JAMA 2008 3 2294 2303 10.1001/jama.299.19.2294 18492971

[B47] Kumar A Ellis P Arabi Y Roberts D Light B Parrillo JE Dodek P Wood G Kumar A Simon D Peters C Ahsan M Chateau D Initiation of inappropriate antimicrobial therapy results in a fivefold reduction of survival in human septic shock Chest 2009 3 1237 1248 10.1378/chest.09-0087 19696123

[B48] Zilberberg MD Carter C Lefebvre P Raut M Vekeman F Duh MS Shorr AF Red blood cell transfusions and the risk of acute respiratory distress syndrome among the critically ill: a cohort study Crit Care 2007 3 R63 10.1186/cc5934 17553147 PMC2206425

[B49] Khan H Belsher J Yilmaz M Afessa B Winters JL Moore SB Hubmayr RD Gajic O Fresh-frozen plasma and platelet transfusions are associated with development of acute lung injury in critically ill medical patients Chest 2007 3 1308 1314 10.1378/chest.06-3048 17400669

[B50] Jia X Malhotra A Saeed M Mark RG Talmor D Risk factors for ARDS in patients receiving mechanical ventilation for > 48 h Chest 2008 3 853 861 10.1378/chest.07-1121 18263691 PMC2628459

[B51] Toy P Popovsky MA Abraham E Ambruso DR Holness LG Kopko PM McFarland JG Nathens AB Silliman CC Stroncek D National Heart, Lung and Blood Institute Working Group on TRALI Transfusion-related acute lung injury: definition and review Crit Care Med 2005 3 4 721 726 10.1097/01.CCM.0000159849.94750.51 15818095

[B52] Popovsky MA Moore SB Diagnostic and pathogenetic considerations in transfusion-related acute lung injury Transfusion 1985 3 6 573 577 10.1046/j.1537-2995.1985.25686071434.x 4071603

[B53] Silliman CC Thurman GW Ambruso DR Stored blood components contain agents that prime the neutrophil NADPH oxidase through the platelet-activating-factor receptor Vox Sang 1992 3 2 133 136 10.1111/j.1423-0410.1992.tb02500.x 1332255

[B54] Densmore TL Goodnough LT Ali S Dynis M Chaplin H Prevalence of HLA sensitization in female apheresis donors Transfusion 1999 3 1 103 106 10.1046/j.1537-2995.1999.39199116901.x 9920173

[B55] Gajic O Yilmaz M Iscimen R Kor DJ Winters JL Moore SB Afessa B Transfusion from male-only versus female donors in critically ill recipients of high plasma volume components Crit Care Med 2007 3 7 1645 1648 10.1097/01.CCM.0000269036.16398.0D 17522583

[B56] Chapman CE Stainsby D Jones H Love E Massey E Win N Navarrete C Lucas G Soni N Morgan C Choo L Cohen H Williamson LM Serious Hazards of Transfusion Steering Group Ten years of hemovigilance reports of transfusion-related acute lung injury in the United Kingdom and the impact of preferential use of male donor plasma Transfusion 2009 3 3 440 452 10.1111/j.1537-2995.2008.01948.x 18980623

[B57] Wright SE Snowden CP Athey SC Leaver AA Clarkson JM Chapman CE Roberts DR Wallis JP Acute lung injury after ruptured abdominal aortic aneurysm repair: the effect of excluding donations from females from the production of fresh frozen plasma Crit Care Med 2008 3 6 1796 1802 10.1097/CCM.0b013e3181743c6e 18496377

[B58] Looney MR Acute lung injury after blood product transfusion: are the times changing? Crit Care Med 2008 3 6 1968 1970 10.1097/CCM.0b013e318176a8b2 18520657

[B59] Vlaar AP Binnekade JM Schultz MJ Juffermans NP Koopman MM Preventing TRALI: ladies first, what follows? Crit Care Med 2008 3 12 3283 3284 19020453 10.1097/CCM.0b013e31818f2f37

[B60] Yazer MH Podlosky L Clarke G Nahirniak SM The effect of prestorage WBC reduction on the rates of febrile nonhemolytic transfusion reactions to platelet concentrates and RBC Transfusion 2004 3 1 10 15 10.1046/j.0041-1132.2003.00518.x 14692961

[B61] Vlaar AP Schultz MJ Juffermans NP Transfusion-related acute lung injury: a change of perspective Neth J Med 2009 3 10 320 326 19915225

[B62] Slofstra SH Groot AP Maris NA Reitsma PH Cate HT Spek CA Inhalation of activated protein C inhibits endotoxin-induced pulmonary inflammation in mice independent of neutrophil recruitment Br J Pharmacol 2006 3 6 740 746 Epub 2006 Oct 3 10.1038/sj.bjp.0706915 17016502 PMC2014647

[B63] Maniatis NA Letsiou E Orfanos SE Kardara M Dimopoulou I Nakos G Lekka ME Roussos C Armaganidis A Kotanidou A Inhaled activated protein C protects mice from ventilator-induced lung injury Crit Care 2010 3 2 R70 Epub 2010 Apr 19 10.1186/cc8976 20403177 PMC2887192

[B64] Kotton DN Ma BY Cardoso WV Sanderson EA Summer RS Williams MC Fine A Bone marrow-derived cells as progenitors of lung alveolar epithelium Development 2001 3 24 5181 5188 11748153 10.1242/dev.128.24.5181

[B65] Chimenti L Luque T Bonsignore MR Ramírez J Navajas D Farré R Pre-treatment with mesenchymal stem cells reduces ventilator-induced lung injury Eur Respir J 2012 3 4 939 948 Epub 2012 Mar 22 10.1183/09031936.00153211 22441745

[B66] Levitt JE Matthay MA Clinical review: Early treatment of acute lung injury - paradigm shift toward prevention and treatment prior to respiratory failure Crit Care 2012 3 3 223 [Epub ahead of print] 10.1186/cc11144 22713281 PMC3580596

